# Effect of Acupuncture on Pneumonia Risk in Stroke Patients using Proton Pump Inhibitors: A Retrospective Case‐Control Study

**DOI:** 10.1002/brb3.70920

**Published:** 2025-09-29

**Authors:** Shaokang Wang, Mi Ou, Haipeng Ban, Tianlong Yin, Yalu Meng, Sihan Sun, Lili Zhang, Yuzheng Du

**Affiliations:** ^1^ First Teaching Hospital of Tianjin University of Traditional Chinese Medicine Tianjin China; ^2^ National Clinical Research Center for Chinese Medicine Acupuncture and Moxibustion Tianjin China; ^3^ Tianjin University of Traditional Chinese Medicine Tianjin China

**Keywords:** Acupuncture, Proton pump inhibitors, Pneumonia, Stroke

## Abstract

**Background:**

Stroke, the second leading cause of death globally, often requires the use of proton pump inhibitors (PPIs) to prevent gastrointestinal bleeding. However, the misuse of PPI can lead to pneumonia, and no current studies have explored the preventive effects of acupuncture on PPI‐induced pneumonia.

**Methods:**

A case‐control study was conducted using data from the Tianjin Healthcare Bigdata Company covering stroke patients on PPI from January 1, 2016 to December 31, 2019. Patients with pneumonia during hospitalization from a stroke diagnosis were assigned to the case group, while those without pneumonia served as the control group. Propensity score matching was applied using a matching threshold set at 0.05. Matching characteristics included age, gender, dementia, Parkinsonism, gastric tube feeding, and oral disease. A logistic regression model was employed to evaluate the impact of acupuncture on the risk of pneumonia in stroke patients using PPI.

**Results:**

In this case‐control study, pneumonia occurred in 6389 (11.11%) stroke patients. Risk factors for pneumonia included suctioning phlegm and hospital stays. When the number of acupuncture sessions was treated as a continuous variable, each additional acupuncture session after the first was associated with a 6% reduction in the risk of pneumonia (odds ratio [OR]: 0.94; 95% confidence intervals [CI]: 0.93–0.95; *p* < 0.001). Multivariable logistic regression revealed that, compared to patients receiving acupuncture less than three times per visit, the risk of pneumonia was significantly reduced by 64% (OR: 0.36; 95% CI: 0.24–0.45; *p* < 0.001) for those receiving acupuncture 7–11 times per visit and by 79% (OR: 0.21; 95% CI: 0.16–0.27; *p* < 0.001) for those receiving acupuncture more than 11 times per visit. The highest quartile pneumonia risk was one‐fifth of the lowest quartile.

**Conclusion:**

In conclusion, this case‐control study suggested that acupuncture may reduce pneumonia risk in stroke patients using PPI. Within a single visit, the best preventive effect was observed with more than 11 acupuncture sessions.

## Graphical abstract

Stroke patients often require the use of proton pump inhibitors (PPIs) to prevent gastrointestinal bleeding. However, the misuse of PPI can lead to pneumonia. This case‐control study suggested that acupuncture may reduce pneumonia risk in stroke patients using PPI.

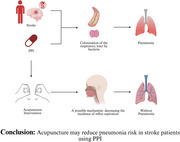

**Abbreviations**
ATP4: H^+^/K^+^‐ATPase
CAP: community‐acquired pneumonia
CI: confidence intervals
GERD: gastroesophageal reflux disease
H2RA: Histamine H2 receptor antagonists
OR: Odds Ratio
PPI: Proton pump inhibitors

## 1 Introduction

As the second leading cause of death worldwide, stroke poses a significant threat to human life and health due to its high incidence and disability rate (Z. Wang et al. 2023). In China, the prevalence of stroke has surpassed that of ischemic heart disease, with an incidence rate of 2.6% (Tu et al. 2023), and continues to rise. This presented a considerable challenge to the country's healthcare system (S. Wu et al. 2019). During the early stages of stroke, patients experience acute stress and require secondary prevention medications (Shim and Kim 2016; Yasuda 2015), which can lead to digestive system symptoms such as vomiting blood and black stools within a week of stroke onset (Ogata et al. 2014), significantly impacting patient prognosis and safety (S. Liu et al. 2022; Yang et al. 2013).

Proton pump inhibitors (PPIs), which target gastric H^+^/K^+^‐ATPase (ATP4), are commonly used to treat gastrointestinal diseases related to gastric acid (El Rouby et al. 2018). However, the misuse of PPI by stroke patients often arises from the need to prevent stress ulcers in nonintensive care unit settings and to mitigate the risk of gastrointestinal bleeding in patients undergoing antiplatelet or anticoagulant therapy (B.‐L. Liu et al. 2013; Savarino et al. 2018). Misuse of PPI can lead to adverse outcomes. Recent evidence has suggested an increased risk of poststroke pneumonia and community‐acquired pneumonia (CAP) associated with PPI usage (Song and Kim 2019; Zirk‐Sadowski et al. 2018). Even after controlling for potential confounders, a moderate correlation between PPI dosage and pneumonia risk remains. The association between short‐term and long‐term PPI use and pneumonia risk is still controversial. Recent research indicated that the mechanism behind PPI‐related pneumonia may involve PPI altering gastric pH, which weakens bacterial growth inhibition and leads to bacterial colonization of the respiratory tract following aspiration of full column reflux, thus triggering pneumonia (Naito et al. 2018).

Acupuncture, as a traditional Chinese therapy, has unique advantages in improving limb function, cognitive function, dysphagia, and other aspects in stroke patients (J. Wang et al. 2020; Xiao et al. 2023; L. Li et al. 2023). Early acupuncture intervention after a stroke aims to prevent further stroke development and complications. The mechanism by which acupuncture may reduce the risk of pneumonia in stroke patients taking PPI could involve repairing the gastrointestinal barrier and decreasing the incidence of reflux aspiration. Therefore, this retrospective case‐control study was to assess the benefits of acupuncture in reducing the risk of pneumonia in stroke patients taking PPI in collaboration with the Tianjin Healthcare Big Data Company.

## 2 Method

### 2.1 Database

This study is a secondary analysis based on the data acquired by Tianjin Healthcare Bigdata Company from 79 secondary and tertiary healthcare institutions in Tianjin, spanning from January 1, 2016 to December 31, 2020. The data encompassed comprehensive outpatient and inpatient records, comprising patient and visit details, medical orders, diagnosis information, and more. Random identification numbers were assigned to patients to protect their privacy. An application for an informed consent exemption was submitted. The study protocol was approved by the Ethics Committee of Tianjin University of Traditional Chinese Medicine (ethics number: TYLL2024[Z]字027) and was conducted following the legal requirements and tenets of the Declaration of Helsinki and its subsequent amendments.

### 2.1 Study Subjects

**FIGURE 1:**
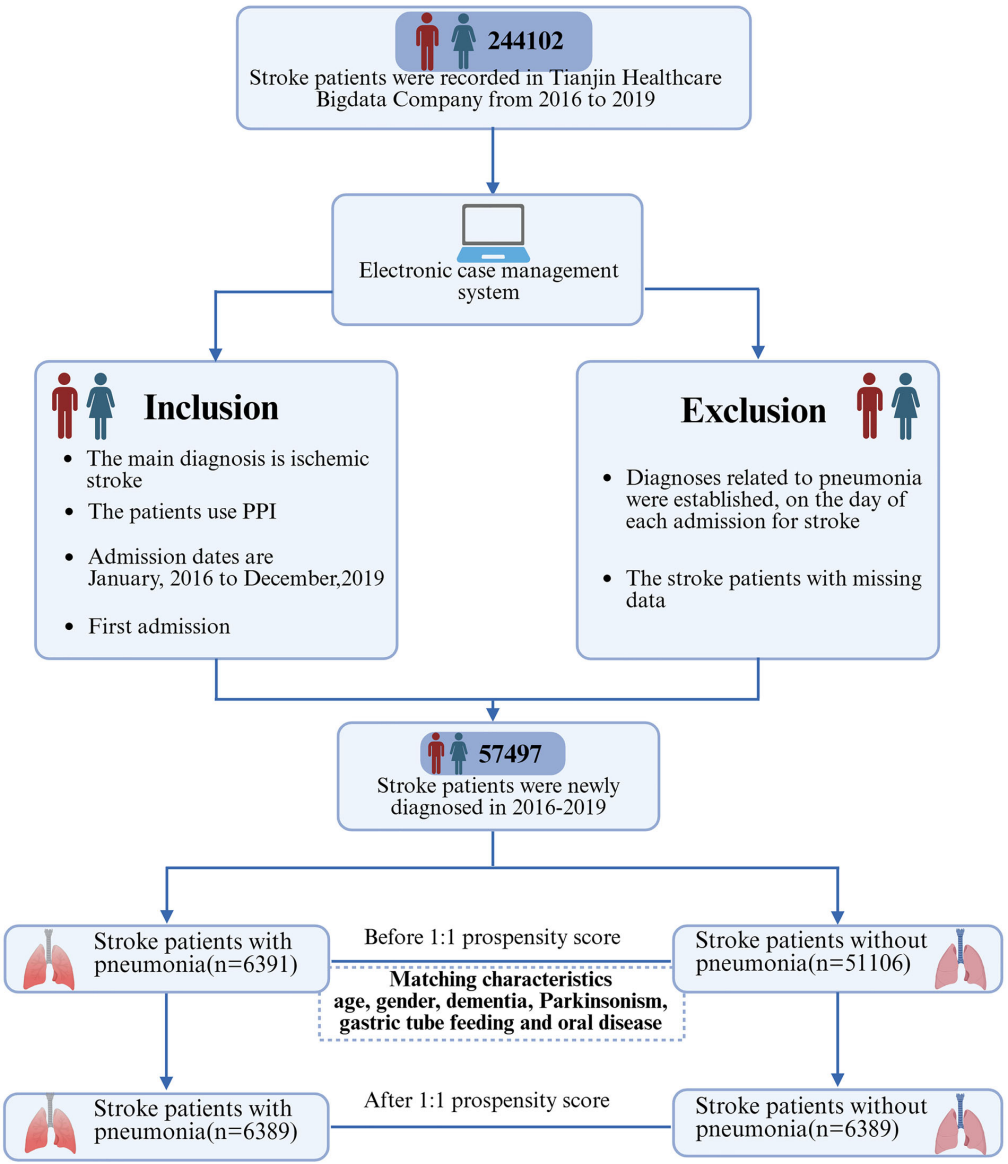
Flowchart of case‐control study.

Our research included patients diagnosed with stroke who were also taking PPI from January 1, 2016 to December 31, 2019, with no restrictions based on age or gender. The first time we accessed the data was on April 26, 2024. Five types of PPI are available in Tianjin: omeprazole, pantoprazole, lansoprazole, esomeprazole, and rabeprazole. This study included only patients who had documented PPI prescriptions during each hospitalization. This study defined pneumonia using ICD codes, with patients diagnosed on admission day with ICD‐10‐J12.xx, 13, 14, 15.xx, 16.xx, 17.xx, or 18.xx classified as having pneumonia. Analysis was conducted for all included patient visits. After applying a series of inclusion and exclusion criteria, 57,497 subjects were included in the study. Patients diagnosed with pneumonia were assigned to the case group. Patients with a single hospitalization and no pneumonia diagnosis were assigned to the control group. To minimize the impact of confounding factors, the propensity score was used to match patients without pneumonia diagnoses, with a matching threshold set at 0.05. Matching characteristics included gastric tube feeding, dementia, Parkinsonism, oral diseases, age, and gender, as illustrated in Figure 1. Gastric‐tube feeding was identified as a risk factor for pneumonia in stroke patients taking PPI. While it is intended to ensure nutritional intake and prevent aspiration in stroke patients, studies suggested that compared to post‐pyloric feeding, gastric tube feeding may increase the risk of pneumonia due to mucosal irritation or injury in the pharyngeal area caused by gastric tube stimulation or improper nasogastric feeding procedure (Y. Liu et al. 2021; Alkhawaja et al. 2015). Additionally, critically ill patients may not be suitable candidates for gastric tube feeding (Huang et al. 2012). Dementia and parkinsonism were also considered as risk factors for pneumonia (Funayama et al. 2023; Won et al. 2020). These conditions often involve dysphagia, an impaired gag reflex, altered consciousness, and an increased risk of aspiration. Furthermore, oral hygiene was associated with respiratory tract infections. Microaspiration of oral bacteria is common, often occurring during sleep (Beal et al. 2004). Dental plaque may harbor respiratory pathogens, and oral microorganisms from periodontal disease may be aspirated into the lungs, triggering pneumonia (Winning et al. 2021).

### 2.2 Study Design

This case‐control study investigated the association between acupuncture treatment and the occurrence of pneumonia in stroke patients taking PPI. The exposure group included various acupuncture modalities, such as body acupuncture, scalp acupuncture, and auricular acupuncture. To explore the effect of the sessions of acupuncture on the risk of pneumonia, we stratified the number of acupuncture treatments per visit. The study endpoint was defined as the occurrence of pneumonia in each visit. For accurate pneumonia diagnosis coding, outpatient information was excluded, and patients diagnosed with pneumonia on the day of hospital admission were not considered. Covariates included age, gender, diabetes, dementia, oral diseases, Parkinsonism, heart failure, gastroesophageal reflux disease (GERD), gastric tube feeding, suctioning phlegm, antipsychotics, statins, histamine H2 receptor antagonists (H2RA), and length of hospital stay. Detailed information on these covariates is provided in Table 1.

**TABLE 1:** Baseline characteristics of the control group and case group before and after matching.

Variable	Unmatched					Matched				
	Control (*n* = 5,1106)		Case(*n* = 6391)		*p* value	Control (*n* = 6389)		Case (*n* = 6389)		*p* value
	*n*	%	*n*	%		*n*	%	*n*	%	
Gender					0.001					0.801
Women	21,921	42.9	2603	40.7		2588	40.5	2603	40.7	
Men	29,185	57.1	3788	59.3						
Age group										
Mean (SD)	66.86 (11.81)		71.35 (12.81)		< 0.001	71.44 (12.67)		71.34 (12.81)		0.662
< 60	11,798	23.1	1007	15.8	< 0.001	922	15.5	1007	15.8	0.935
60–75	24,433	47.8	2367	37.0		2372	37.1	2367	37.0	
≥ 75	14,875	29.1	3017	47.2		3025	47.3	3015	47.2	
Acupuncture	10,912	21.4	1050	16.4	< 0.001	1677	26.2	1050	16.4	< 0.001
Q1	2561	23.5	429	40.9	< 0.001	364	21.7	429	40.9	< 0.001
Q2	2723	25.0	268	25.5		336	20.0	268	25.5	
Q3	2802	25.7	188	17.9		505	30.1	188	17.9	
Q4	2826	25.9	165	15.7		472	28.1	165	15.7	
Diabetes	22,106	43.3	2228	34.9	< 0.001	2655	41.6	2228	34.9	< 0.001
Dementia	2397	4.7	70	1.1	< 0.001	85	1.3	70	1.1	0.258
Oral diseases	576	1.1	33	0.5	< 0.001	20	0.3	33	0.5	0.099
Parkinson	948	1.9	110	1.7	0.483	98	1.5	110	1.7	0.442
Heart failure	8421	16.5	1401	21.9	< 0.001	1427	22.3	1400	21.9	0.579
GERD	1527	3.0	59	0.9	< 0.001	148	2.3	59	0.9	< 0.001
Gastric tube feeding	7457	14.6	3784	59.2	< 0.001	3779	59.1	3782	59.2	0.971
Suctioning phlegm	13,139	25.7	3557	59.7	< 0.001	3199	50.1	3555	55.6	< 0.001
Antipsychotics	1554	3.0	420	6.6	< 0.001	377	5.9	420	6.6	0.124
Statin	41,251	80.7	3391	53.1	< 0.001	4803	75.2	3390	53.1	< 0.001
H2RA	222	0.4	36	0.6	0.176	23	0.4	36	0.6	0.117
Length of hospital time					< 0.001					< 0.001
Mean (SD)	13.29 (15.77)		19.61 (26.90)			17.57 (29.49)		19.61 (26.90)		
≤ 14 days	32,286	63.2	2806	43.9	< 0.001	3562	55.8	2805	43.9	< 0.001
15–30 days	17,1113	33.5	2713	42.5		2227	34.9	2712	42.4	
> 30 days	1707	3.3	872	13.6		599	9.4	872	13.6	

*Notes*: Q1–Q4 under Acupuncture are according to the number of acupuncture quarterback partitions. *p* value < 0.05 was considered statistically significant.

Abbreviations: GERD, gastroesophageal reflux disease; H2RA: Histamine H_2_ receptor antagonists.

### 2.3 Statistical Analysis

Categorical variables were summarized using numbers and percentages, and differences between groups were assessed using Chi‐square tests. Continuous variables were first tested for normal distribution with the Kolmogorov–Smirnov test and were described with mean ± SD. Comparisons between groups were conducted using one‐way ANOVA. Multivariable logistic regression balanced intergroup differences and was applied to adjust for potential confounders such as underlying diseases and treatments that may affect pneumonia risk. Odds ratios (OR) with 95% confidence intervals (CI) were calculated to identify risk factors and evaluate their effects. A *p* value < 0.05 was considered statistically significant. Statistical analyses were performed using R version 4.2.1.

## 3 Result

We performed propensity score matching for variables including age, gender, dementia, Parkinsonism, gastric tube feeding, and oral disease. This matched cohort comprised a case group (*n* = 6389) and the control group (*n* = 6389). Baseline characteristics of the participants are summarized in Table 1.

Several factors were associated with pneumonia among stroke patients using PPI but without pneumonia, including age, acupuncture, diabetes, heart failure, GERD, suctioning phlegm, antipsychotics, statins, H2RA, and length of hospital stay. After adjusting for these variables, suctioning phlegm and longer hospital stays were found to increase the risk of pneumonia. In the univariate analysis, age was not a risk factor for pneumonia induced by PPI use in stroke patients. In the multivariate analysis, diabetes was a protective factor. Conversely, acupuncture was found to be a protective factor against pneumonia (OR: 0.54, 95% CI: 0.50–0.60). Hospitalization for 15–30 days (OR: 1.55, 95% CI: 1.44–1.67) and for more than 30 days (OR: 1.85, 95% CI: 1.65–2.07) both elevated the risk of pneumonia. Detailed information is shown in Table 2

**TABLE 2:** Odds ratio and 95% confidence intervals of pneumonia with acupuncture and covariates.

	Univariable analysis		Multivariable analysis	
	OR (95% CI)	*p* Value	OR (95% CI)	*p* Value
Age group				
< 60	ref		—	
60–74	0.98 (0.89–1.09)	0.748	—	
≥ 75	0.98 (0.89–1.09)	0.723	—	
Acupuncture	0.55 (0.51–0.60)	< 0.001	0.54 (0.50–0.60)	< 0.001
Diabetes	0.75 (0.70–0.81)	< 0.001	0.79 (0.73–0.85)	< 0.001
Heart failure	0.98 (0.90–1.06)	0.565	—	
GERD	0.39 (0.29–0.53)	< 0.001	0.42 (0.30–0.57)	< 0.001
Suctioning phlegm	1.25 (1.17–1.34)	< 0.001	1.11 (1.03–1.20)	0.006
Antipsychotics	1.12 (0.97–1.30)	0.116	—	
Statin	0.37 (0.35–0.40)	< 0.001	0.42 (0.39–0.45)	< 0.001
H2RA	1.57 (0.93–2.68)	0.0925	—	
Length of hospital time				
≤ 14 days	ref		ref	
15–30 days	1.55 (1.44–1.67)	< 0.001	1.64 (1.52–1.78)	< 0.001
> 30 days	1.85 (1.65–2.07)	< 0.001	1.58 (1.40–1.79)	< 0.001

*Note*: *p* < 0.05 was considered statistically significant.

Abbreviations: GERD, gastroesophageal reflux disease; H2RA, Histamine H_2_ receptor antagonists;

In the univariable logistic analysis, when the number of acupuncture sessions was treated as a continuous variable, each additional acupuncture session after the first was associated with a 6% reduction in risk (OR: 0.94, 95% CI: 0.93–0.95). When the number of acupuncture sessions was treated as a categorical variable classified by quartiles, they were Q1: 1–3 sessions, Q2: 4–6 sessions, Q3: 7–11 sessions, and Q4: > 11 sessions. When acupuncture sessions were categorized and Q1 was used as the reference, Q2, Q3, and Q4 were significantly correlated with a lower risk of pneumonia (Q2, OR: 0.68, 95% CI: 0.55–0.84; Q3, OR: 0.32, 95% CI: 0.25–0.39; Q4, OR: 0.30, 95% CI: 0.24–0.37). Even after adjusting for length of hospital stay, acupuncture remained significantly linked to a reduced risk of pneumonia (Q2, OR: 0.67, 95% CI: 0.54–0.83; Q3, OR: 0.32, 95% CI: 0.26–0.40; Q4, OR: 0.27, 95% CI: 0.21–0.33). In the multivariable logistic analysis, Q3 and Q4 were significantly associated with a reduced risk of pneumonia (Q3, OR: 0.36, 95% CI: 0.24–0.45; Q4, OR: 0.21, 95% CI: 0.16–0.27). Detailed information is shown in Table 3.

**TABLE 3:** Odds ratio and 95% confidence intervals of pneumonia with acupuncture times and quartile interval.

Exposure	Univariable analysis		Model 1		Multivariable analysis	
	OR (95% CI)	*p* Value	OR (95% CI)	*p* Value	OR (95% CI)	*p* Value
Acupuncture times	0.94 (0.93–0.95)	< 0.001	0.94 (0.93–0.94)	< 0.001	0.93 (0.92–0.94)	< 0.001
Q1	reference		reference		reference	
Q2	0.68 (0.55–0.84)	< 0.001	0.67 (0.54–0.83)	< 0.001	0.81 (0.64–1.01)	0.065
Q3	0.32 (0.25–0.39)	< 0.001	0.32 (0.26–0.40)	< 0.001	0.36 (0.24–0.45)	< 0.001
Q4	0.30 (0.24–0.37)	< 0.001	0.27 (0.21–0.33)	< 0.001	0.21 (0.16–0.27)	< 0.001

*Note*: Model 1: Multivariate analysis after adjusting length of stay. Q1–Q4 under Acupuncture is according to the number of acupuncture quarterback partitions. *p* < 0.05 was considered statistically significant.

## 4 Discussion

To our knowledge, this study is the first to investigate the impact of acupuncture on pneumonia risk in stroke patients using PPI, based on a large‐scale case‐control study. The results indicated that acupuncture can reduce pneumonia risk associated with PPI usage in stroke patients, acting as a protective factor. Within a single visit, the best preventive effect occurred with acupuncture times exceeding 11 times. The cumulative effect of acupuncture sessions on the prevention of pneumonia remains a topic that warrants further investigation.

The use of PPI was often deemed inappropriate (Pasina et al. 2011; Jarchow‐Macdonald and Mangoni 2013). In stroke patients, PPIs were commonly administered preemptively to prevent gastrointestinal bleeding from ulcers. However, misuse of PPI may trigger a range of adverse reactions, including Clostridium difficile infection, cardiovascular diseases, and fractures (Xun et al. 2022; Melloni et al. 2015; Poly et al. 2019). Some observational studies suggested an increased risk of poststroke pneumonia in patients using PPI and H2RA, with a higher pneumonia risk associated with high‐dose PPI (Song and Kim 2019). Sarkar et al. (2008) found a significant correlation between PPI usage duration and the risk of CAP, with the highest risk occurring within 30 days of use. Additionally, dementia patients should use PPI cautiously (Ho et al. 2017). Mechanistically, there was still no definitive conclusion regarding the relationship between PPI usage and pneumonia. Studies indicated a significant correlation between the bacteria abundance in the stomach and duodenum and gastric pH (Thorens et al. 1996). An increase in pH led to an increase in bacterial abundance in the stomach, particularly acid‐sensitive bacteria such as streptococci and staphylococci (Rosen et al. 2014; Williams and McColl 2006). Suctioning phlegm is a common practice in the daily care of stroke patients and can also be used diagnostically to assist doctors to identify pathogens, diagnose pneumonia types, and determine the necessity of antibiotics (Sachdev et al. 2010). However, our findings indicated that phlegm suctioning could increase the risk of pneumonia, potentially due to inadequate adherence to aseptic techniques during the procedure. Additionally, the diagnostic use of phlegm suctioning needs careful consideration regarding its impact on pneumonia risk. Our study suggested that diabetes might reduce the risk of pneumonia associated with PPI use in stroke patients, contrary to previous research findings (Ho et al. 2017; Jiménez‐Trujillo et al. 2017). This could be attributed to diabetic patients being more vigilant about managing their blood sugar levels. Diabetes patients may be more concerned about blood sugar levels as a result. Regardless of diabetes status, You et al. (2019) demonstrated that higher fasting blood glucose levels upon admission are more likely to increase the risk of pneumonia in acute ischemic stroke patients. Our study indicated that elevated blood glucose levels, rather than diabetes itself, may be a significant risk factor for pneumonia, possibly due to stress‐induced hyperglycemia at the time of admission (Roberts et al. 2023).

Some observational studies suggested microbial community exchange between the stomach and lungs with species overlap between the microbial communities in the stomach and oral cavity. This indicates that the gastric microbiota induced by aspiration of full column reflux may influence the upper respiratory tract microbiota (Rosen et al. 2015; Segal et al. 2006). In stroke patients, PPI elevated gastric pH, significantly increasing streptococci and enterococci. Following aspiration of full column reflux, these bacteria may become potential pathogens for upper respiratory tract infections and pneumonia. Additionally, changes in the symbiotic bacteria between the stomach and lungs may alter the microbial environment, potentially increasing the risk of pulmonary pathogen infection and reducing the benefit of acid‐suppressing medications (Rosen et al. 2014; Sakwinska et al. 2014). An experimental study suggested that PPI inhibited ATP4 function in the mature mucociliary epithelium, impairing the function of the mucociliary epithelium in the airways and increasing susceptibility to pulmonary infections (Walentek et al. 2015).

Acupuncture has been shown to help prevent infections. A meta‐analysis showed that in patients with swallowing difficulties due to Parkinson's disease, acupuncture significantly reduced the incidence of pulmonary infections compared to those who did not receive acupuncture (J. Wu et al. 2023). Additionally, a cohort study demonstrated that acupuncture reduced the risk of pneumonia in stroke patients, especially in women and younger individuals (Chang et al. 2018). However, there is currently no research exploring the preventive effects of acupuncture on pneumonia caused by PPI use in stroke patients. Our finding indicated that each additional acupuncture session during a single visit was associated with a 6% reduction in pneumonia risk, with the most significant preventive effect occurring after more than 11 sessions. Previous studies have shown that four or more acupuncture sessions were moderately correlated with a reduction in pneumonia risk (Chang et al. 2018), which aligns with our results. Our study indicated that the more acupuncture sessions received, the lower the risk of pneumonia. Multivariate analysis showed that the protective effect of > 11 acupuncture sessions during a single hospitalization remained robust (OR = 0.21) after controlling for comorbidities. When further adjusting for length of stay, the OR increased to 0.27. Model 1 indicated no difference in hospitalization duration between the two groups. The positive correlation between length of stay and number of acupuncture sessions suggested that > 11 sessions per hospitalization might require a longer inpatient period to complete treatment. Evidence shows that prolonged hospitalization alone increases pneumonia risk due to cumulative exposure to hospital pathogens (Roberts et al. 2023). Extended length of stay is an independent risk factor for pneumonia (Table 2: 15–30 days OR: 1.55, > 30 days OR: 1.85) because the risk of nosocomial infections escalates with hospitalization duration.

There were several proposed mechanisms regarding the potential pathways through which acupuncture may affect pneumonia induced by PPI use. First, some studies suggested that the gastrointestinal barrier is an essential component of gastrointestinal defense, and acupuncture may help repair this barrier through neuroendocrine pathways and anti‐inflammatory effects (H. Li 2015). In patients with functional gastrointestinal disorders, acupuncture at CV‐12 may help restore the gastrointestinal barrier via the somatic sympathetic nervous pathway (Takahashi 2006). Evidence suggests that acupuncture can alleviate damage to the gastrointestinal mucosal barrier caused by stress and metabolic changes in stroke patients, thereby improving gastrointestinal function (Qin et al. 2022). Additionally, it can regulate the intestinal flora structure in patients with ischemic stroke, mitigate inflammatory responses, and accelerate the repair of the gastrointestinal barrier (Jiang et al. 2024). The gut–brain axis links the central nervous system and the gastrointestinal tract, regulating gastrointestinal function (Bonaz and Sabate 2009). Acupuncture has bidirectional regulatory effects and may improve gastric secretion in gastrointestinal systems with insufficient gastric acid, promoting gastric acid secretion and maintaining an acid‐base balance in the digestive system (Rehfeld 2021; Gao et al. 2007; Dickman et al. 2007). Second, while studies indicated that PPI could improve reflux diseases caused by excessive gastric acid, some viewpoints suggested that the association between PPI use and pneumonia may be related to reflux aspiration. Dickman et al. (2007) suggested that acupuncture combined with PPI might be more effective in treating GERD when standard doses of PPI fail to control symptoms. A randomized controlled trial demonstrated that thread‐embedded acupuncture effectively alleviated gastric burning and reflux symptoms in GERD patients, resulting in lower scores on the Gastroesophageal Reflux Disease Questionnaire (Trinh et al. 2023). According to traditional Chinese medicine theory, PC6, SP4, and DU20 were commonly used acupoints to alleviate symptoms like nausea, vomiting, and abdominal distension, providing a potential pathway for reflux inhibition (Ouyang 2007). Acupuncture can enhance the coordination of fine movements in the tongue and pharyngeal muscles, increase the activity of oropharyngeal muscle groups, and thereby reduce aspiration (H. Li et al. 2024).

This study is the first to investigate the impact of acupuncture on the risk of pneumonia in stroke patients using PPIs, based on a large‐scale case‐control study. The database contains extensive information on patients' inpatient and outpatient records, which can provide a reliable guarantee for the derivation of evidence‐based evidence. However, this study has several limitations. First, due to the high number of missing values of PPI dosage in the database, we did not explore the association between acupuncture and pneumonia risk related to PPI dosage. There was a dose‐dependent relationship between PPI use and pneumonia risk, with the defined daily dose representing the average maintenance dose recommended by the World Health Organization for adults as the primary indication for this medication (Sinnott et al. 2016). High doses of PPI can increase the risk of pneumonia. Second, our study did not investigate the effects of individual acupoints or combinations of acupoints on pneumonia. In practice, acupoint selection and techniques vary among acupuncturists based on the severity of the stroke and different complications. Third, patients may also receive other traditional Chinese medicine therapies, such as moxibustion and herbal medicine, which could overestimate acupuncture's efficacy. Finally, due to difficulties accurately labeling this field, we did not include dysphagia as a covariate in this study. Dysphagia is often considered an important confounding factor in PPI‐induced pneumonia.

In conclusion, a case‐control study suggested that acupuncture might reduce pneumonia risk in stroke patients using PPI, with the most significant preventive effect occurring after more than 11 acupuncture sessions with a single visit. Therefore, it is crucial in clinical practice to recognize the risks associated with PPI misuse in stroke patients and to consider the preventive benefits of acupuncture treatment.

## Author Contributions

**Shaokang Wang**: writing – original draft. **Mi Ou**: writing – original draft, data curation, methodology. **Haipeng Ban**: writing – review and editing, conceptualization. **Tianlong Yin**: writing – review and editing, conceptualization. **Yalu Meng**: writing – review and editing, formal analysis. **Sihan Sun**: writing—review and editing, formal analysis. **Lili Zhang**: writing—review and editing, formal analysis. **Yuzheng Du**: writing—review and editing, supervision, resources, funding acquisition.

## Ethics Statement

An application for informed consent exemption was submitted. The study protocol was approved by the Ethics Committee of Tianjin University of Traditional Chinese Medicine (ethics number: TYLL2024[Z]字027) and was conducted following the legal requirements and tenets of the Declaration of Helsinki and its subsequent amendments.

## Conflicts of Interest

The authors declare no conflicts of interest.

## Peer Review

The peer review history for this article is available at https://publons.com/publon/10.1002/brb3.70920

## Data Availability

Data are available upon reasonable request. The datasets generated and/or analyzed during the current study are available from the corresponding author on reasonable request.
